# Is an anteromedial minimally invasive approach for middle and distal third humeral fractures feasible? A cadaveric study and clinical case series

**DOI:** 10.1186/s10195-023-00684-9

**Published:** 2023-02-10

**Authors:** Jing Yang, Zhenxing Yang, Dapeng Liu, Zhanxin Lu, Cheng Tao, Tang Liu

**Affiliations:** 1 Department of Orthopedics, The Second Xiangya Hospital of Central South University, Changsha, 410011 Hunan China; 2 Department of Orthopedics, The Fifth Affiliated Hospital of Xinjiang Medical University, Urumqi, 830000 Xinjiang China

**Keywords:** Humeral shaft fractures, Distal third, MIPPO, Anteromedial, Neurovascular injury

## Abstract

**Background:**

Iatrogenic injury to the radial nerve is a risk in surgical treatment for extraarticular fractures of the middle and distal third of the humerus. We aimed to investigate the safety, feasibility and advantages of minimally invasive percutaneous plate osteosynthesis (MIPPO) via an anteromedial approach in the treatment of middle and middle-distal humeral fractures and to evaluate proximity to neurovascular structures.

**Materials and methods:**

In 2016, 13 adult cadaver arms were used to simulate a minimally invasive surgical approach to the anteromedial humerus followed by fixation with a locking compression plate (LCP), and several sets of anatomical data were measured to clarify the possible risk of iatrogenic vascular and nerve injury in this surgical approach. Then, a case series study of 12 patients with humeral fractures who were treated with this surgical approach was conducted between 2017 and 2020.

**Results:**

The average humeral length was 29.22 ± 1.62 cm, the average width of the medial epicondyle of the humerus was 1.31 ± 0.17 cm, and the average distance from the vertex of the medial epicondyle to the median nerve was 2.96 ± 1.62 cm. Furthermore, the safe area for distal humeral screw placement was 6.28 ± 0.39 cm, and the average distance from the tip of the distal end of the screw in the medial epicondyle to the ulnar nerve was 1.7 ± 1.25 mm. None of the 12 patients had nerve damage or an incisional infection after the operation.

**Conclusions:**

The new approach was performed as described, and no cases of iatrogenic nerve palsy occurred. This approach can be used as an alternative for the treatment of extraarticular fractures of the middle and distal thirds of the humerus.

*Level of Evidence:* Level IV, therapeutic study.

## Introduction

Open reduction with internal fixation (ORIF) is a common treatment method. However, these methods involve great trauma and a risk of iatrogenic radial nerve injury of 5.1–31.3% [1, 2].

Minimally invasive percutaneous plate osteosynthesis (MIPPO) is emerging as an effective alternative. In the treatment of humeral shaft fractures, three surgical approaches, namely, the anterior approach, anterolateral approach and posterior approach, are commonly used. Each has its advantages and disadvantages. To the best of our knowledge, no physician has evaluated a minimally invasive anteromedial approach for the treatment of middle and distal humeral fractures or conducted related case series studies.

We hypothesize that the use of a minimally invasive anteromedial approach for internal fixation in the treatment of middle and distal third humeral extraarticular fractures is feasible, that it does not carry the risk of neurovascular injury, and that this approach can be used to treat middle and distal third humeral fractures and evaluate proximity to neurovascular structures.

## Materials and methods

### Cadaveric verification

The study was approved by the hospital's ethics committee. In all, 13 adult cadaver arms (8 left arms and 5 right arms) were used. The donors had no history of deformity or upper extremity surgery. First, the medial epicondyle was palpated, and the skin was cut 3–4 cm proximal to the medial edge of the intermuscular sulcus of the biceps. Then, the gap between the biceps and triceps was determined, the basal vein and the medial cutaneous nerve of the forearm were identified and protected, and the brachial muscle fascia was cut to expose the anteromedial surface of the distal humerus. The brachialis muscle was retracted laterally to protect the anterior blood vessels and nerves, while the triceps brachii protected the posterior ulnar nerve. Then, the pronator teres muscle was retracted medially to expose the upper part of the medial condyle of the humerus and allow steel plate insertion.

The locking compression plate (LCP) was placed on the skin, and the location of the incision at the proximal humerus was determined. By palpation, the gap between the proximal end of the proximal biceps and the deltoid muscle was determined. After the skin was cut, the long head of the biceps brachii tendon was identified, the long head of the biceps brachii tendon was retracted to the outside, and dissection was continued down to the proximal anteromedial surface of the humeral shaft.

**Fig. 1:**
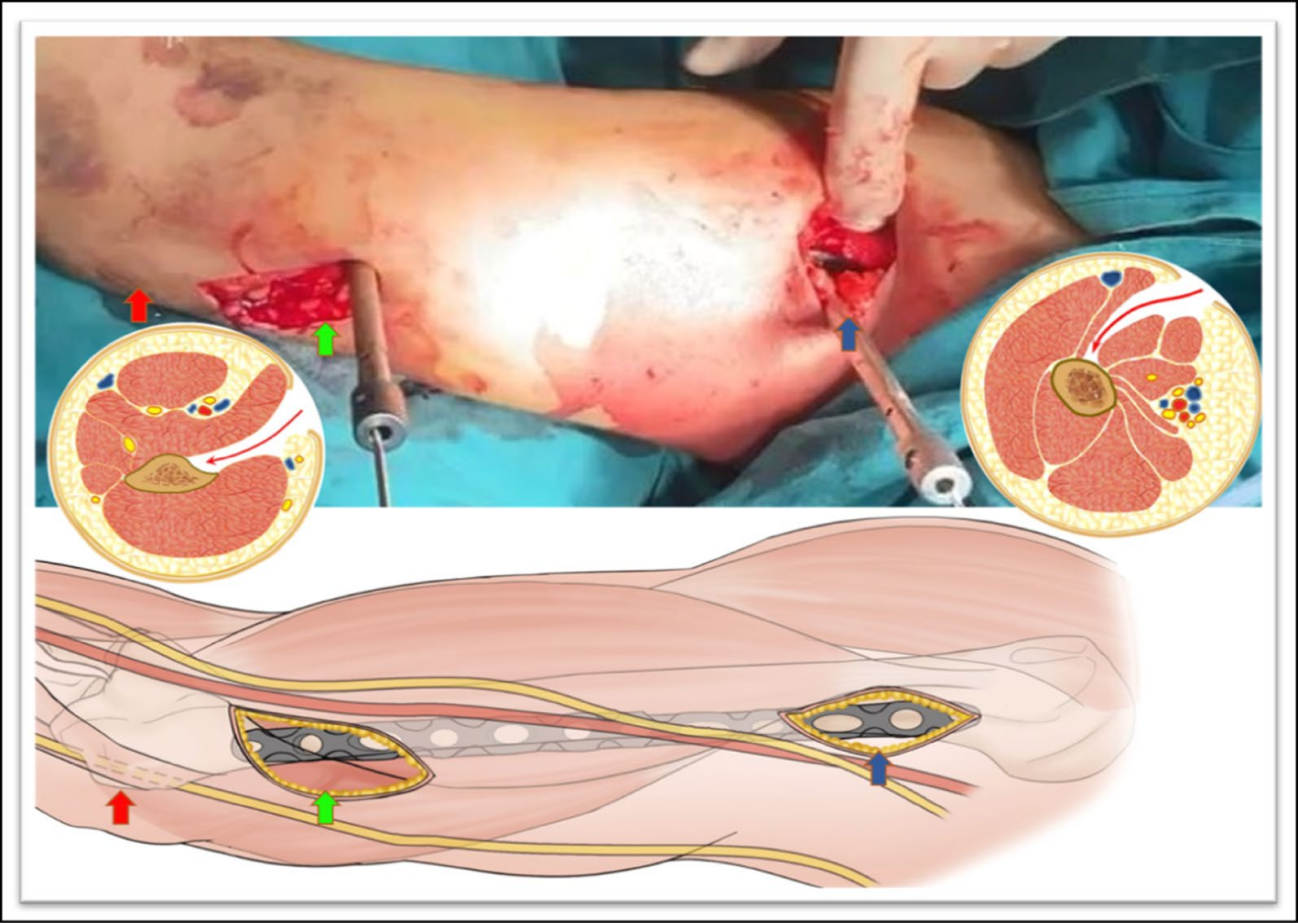
This figure shows proximal (*blue arrows*) and distal (*green arrows*) incisions of the right arm and diagrams of the plane of dissection.* Red arrows* indicate the the medial epicondyle

The 12-hole LCP was inserted with a locking drill sleeve through the distal incision. Under the brachialis, a soft tissue tunnel was established on the anteromedial side of the humerus. The plate was inserted and positioned with two locking sleeves on the anteromedial surface of the humerus (Fig. 1).

**Fig. 2:**
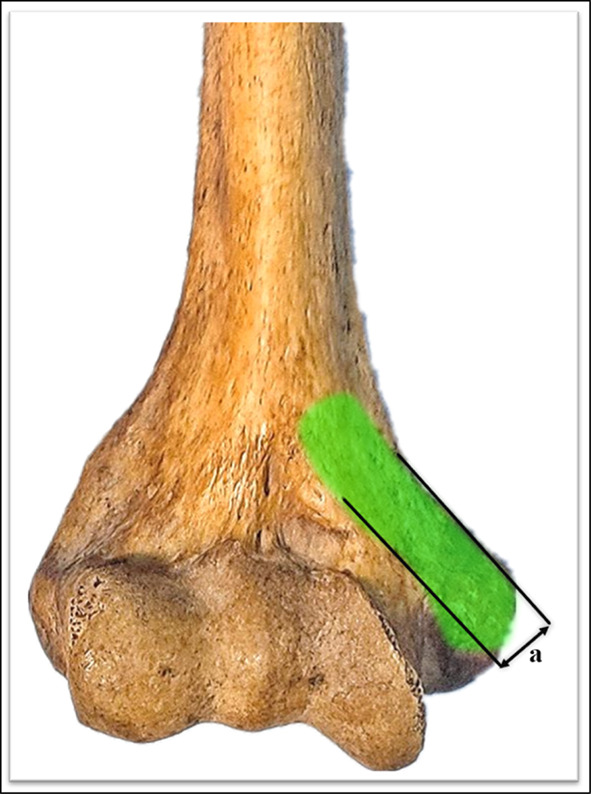
This figure shows the distance from the medial condyle to the base of the coronal fossa (*a*). The* green area* represents the single-cortical fixation area for screws in the medial epicondyle region of the humerus

**Fig. 3:**
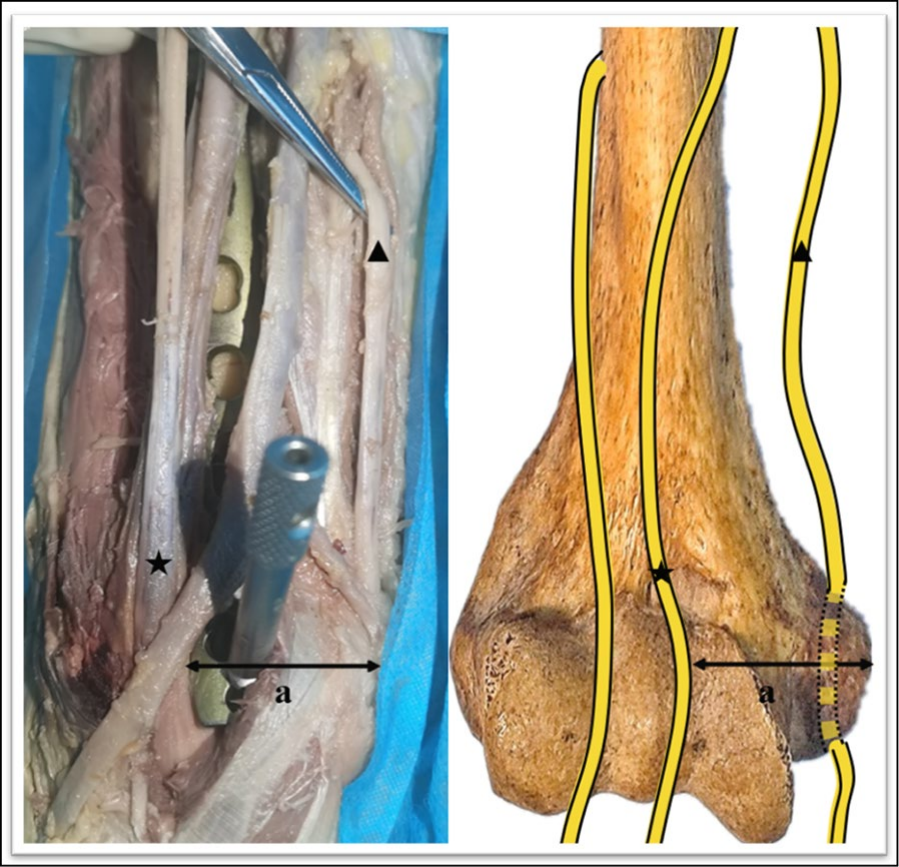
This figure shows the vertical distance from the vertex of the medial epicondyle to the median nerve (*a*). The* pentagrams* indicate the median nerves.* Triangles* indicate ulnar nerves

**Fig. 4:**
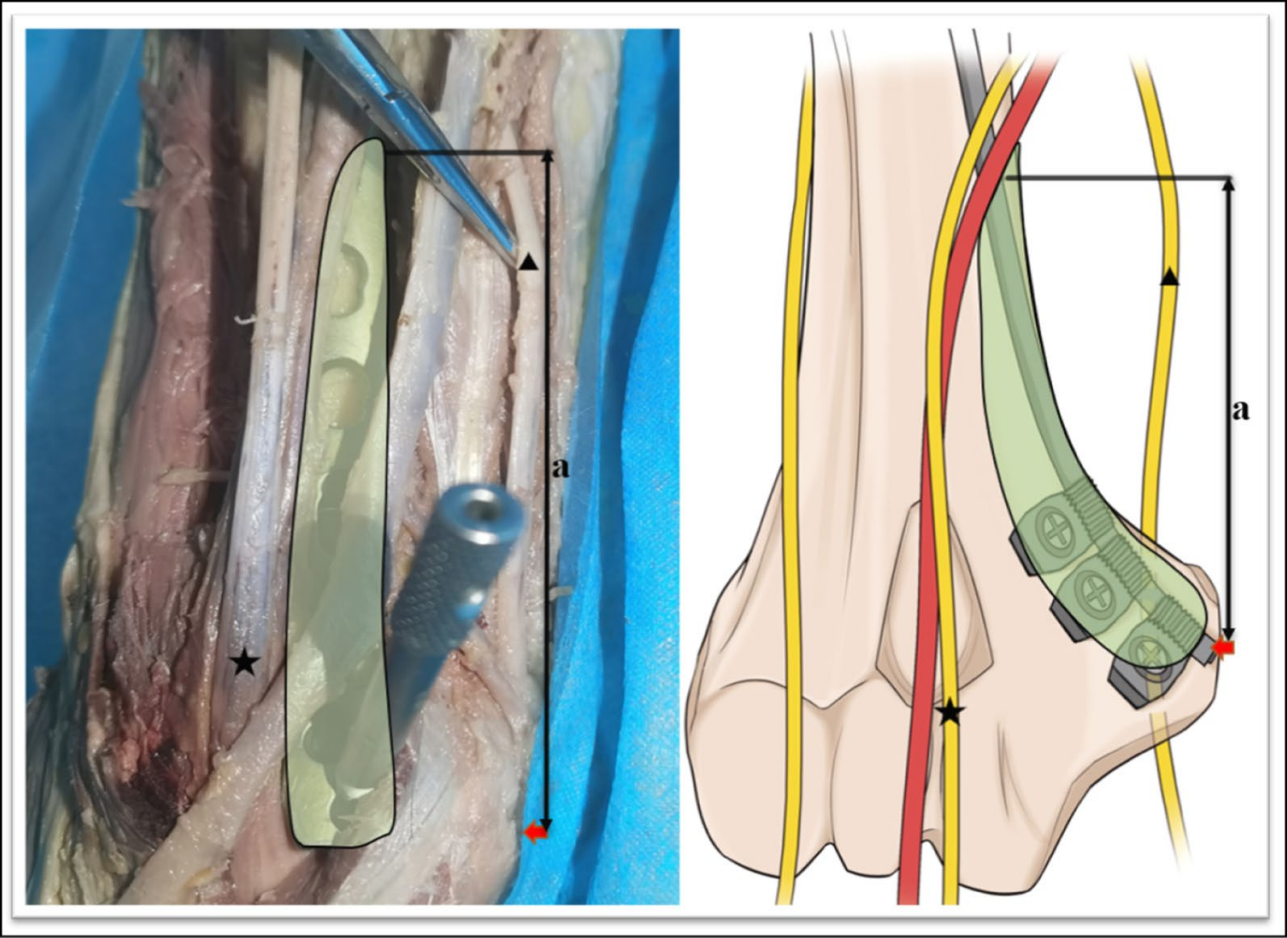
This figure shows the distance from the vertex of the epicondyle to the intersection of the median nerve and the distal end of the underlying steel plate (*a*).* Green areas* represent the safe area where the screw is placed at the distal end. The* pentagrams* indicate the median nerves. The* triangles* indicate the ulnar nerves. The* red arrows* indicate the medial epicondyle

**Fig. 5:**
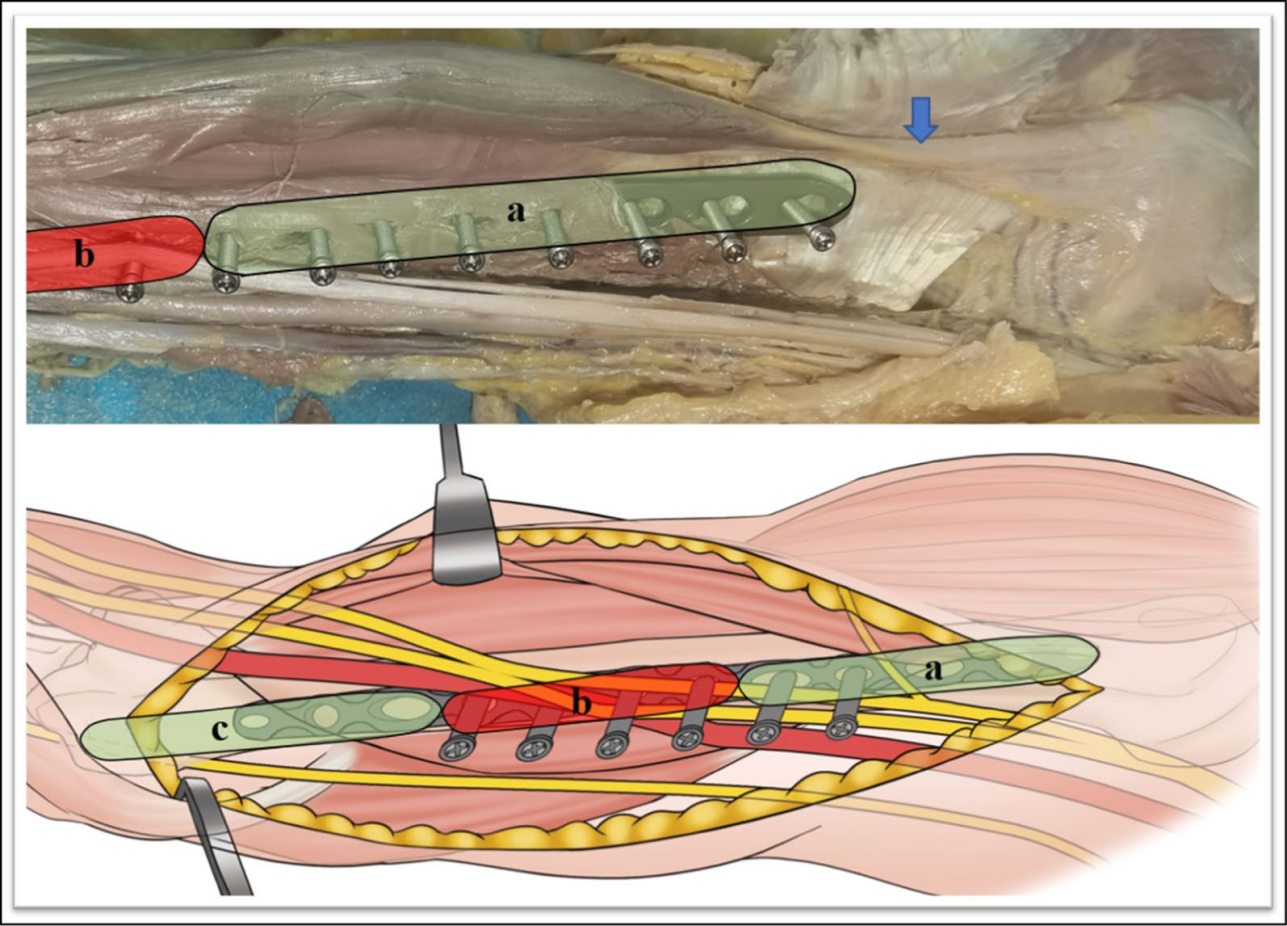
This figure shows the distance, parallel to the long axis of the humerus, from the medial base of the humeral head to the intersection of the median nerve and the proximal end of the underlying steel plate (*a*). The* green areas* indicate the safe area for screw placement (*a* and *c*). The* blue arrow* indicates the long head of the brachii tendon. Percutaneous screw fixation is not suitable between the distal and proximal incisions (*b*; the* red areas*)

**Fig. 6:**
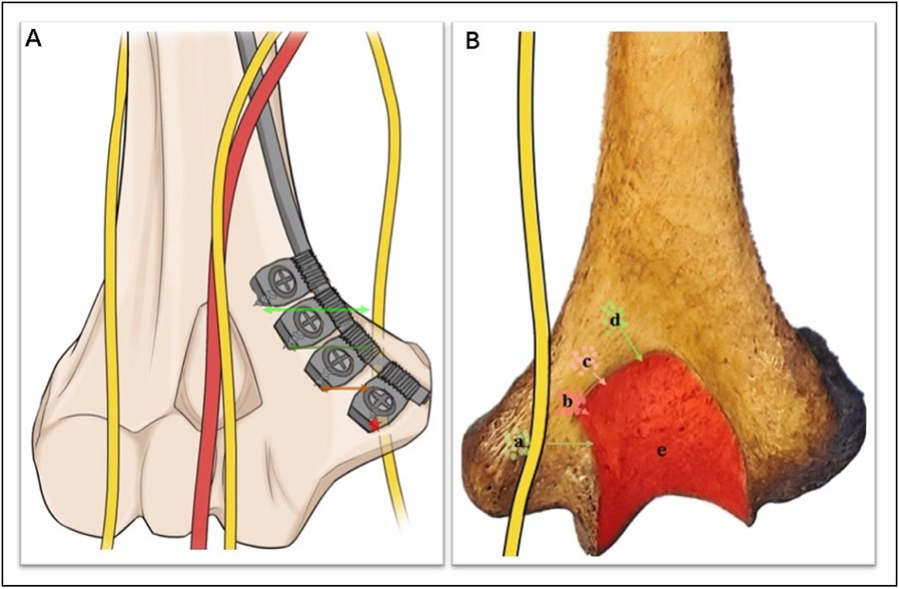
**A** The distal end of the plate with four screws crosses the distance between the tip of the bicortical cortex and the ulnar nerve. **B** The distance between the olecranon fossa and the tip of the four screws (regions* a*–*d*) at the distal end of the plate in the medial humeral epicondyle. The* red area* (*e*) represents the olecranon fossa and the articular surface of the olecranon

**Fig. 7:**
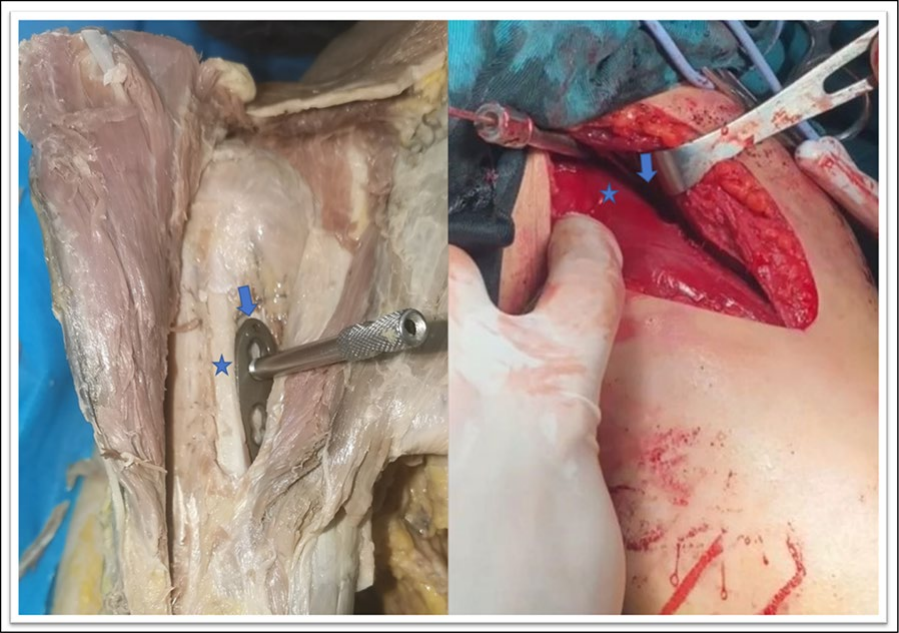
This figure shows the positional relationship between the plate and the biceps brachii in the proximal incision. The* blue pentagrams* represent the long head of the brachii tendon. The* blue arrow* represents the LCP

The relevant measurements were as follows: (1) the mean distance from the medial condyle to the base of the coronal fossa (each specimen was measured three times, and the average was calculated) (Fig. 2); (2) the vertical distance from the vertex of the medial epicondyle to the median nerve (Fig. 3); (3) the length of the humerus from the greater tuberosity to the apex of the lateral condyle; (4) the distance from the medial epicondyle to the lateral epicondyle of the humerus; (5) the distance from the vertex of the epicondyle parallel to the long axis of the humerus to the intersection of the median nerve and the distal end of the underlying steel plate, namely, the safe area for distal screw placement (Fig. 4); (6) parallel to the long axis of the humerus, the distance from the medial base of the humeral head to the intersection of the median nerve and the proximal end of the underlying steel plate, namely, the safe area for proximal screw placement (Fig. 5); (7) located on the medial epicondyle, the distance between the tip of the bicortical screw and the ulnar nerve, which is crossed by the distal end of the plate with four screws; and (8) the distance between the olecranon fossa and the tip of the four screws at the distal end of the plate in the medial humeral epicondyle (Fig. 6 and Tables 1, 2, 3).

**Table 1:** Relevant anatomical data obtained at the time of dissection

Measurement area	Specimen number/left (L) or right (R) side												
	No. 1 R	No. 2 R	No. 3 L	No. 4 L	No. 5 L	No. 6 R	No. 7 L	No. 8 R	No. 9 L	No. 10 L	No. 11 L	No. 12 R	No. 13 L
1. The distance from the medial condyle to the base of the coronal fossa (cm)	1.40	1.53	1.40	1.39	1.47	1.42	1.52	1.28	1.23	1.10	1.17	1.10	1.07
2. The vertical distance from the vertex of the medial epicondyle to the median nerve (cm)	3.1	3.0	3.3	3.2	3.0	3.4	3.1	2.9	2.9	2.6	2.9	2.8	2.3
3. The length of humerus (cm)	29.3	28.5	29.5	31.5	27.5	28.5	27.0	31.0	32.5	29.0	28.6	27.5	29.5
4. The distance from the medial epicondyle to the lateral epicondyle of the humerus (cm)	6.48	6.68	6.78	6.73	6.50	6.50	6.45	6.01	5.90	5.55	5.20	5.21	5.92
5. The distance from the vertex of the epicondyle to the intersection of the median nerve and the distal end of the underlying steel plate (cm)	6.8	6.4	6.2	6.5	6.3	5.7	6.7	6.4	6.2	6.9	6.1	5.8	5.7
6. The distance from the medial base of the humeral head to the intersection of the median nerve and the proximal end of the underlying steel plate. (cm)	9.2	9.8	9.0	10.6	8.8	8.6	7.0	9.2	10.1	8.6	8.5	7.6	8.5

**Table 2:** Located on the medial epicondyle, the distances (mm) between the tips of the bicortical screws and the ulnar nerve, which is crossed by the distal end of the plate with four screws

Measurement area	Specimen number/left (L) or right (R) side												
	No. 1 R	No. 2 R	No. 3 L	No. 4 L	No. 5 L	No. 6 R	No. 7 L	No. 8 R	No. 9 L	No. 10 L	No. 11 L	No. 12 R	No. 13 L
1. First distal screw of the plate	0	2	3	2	1	2	3	2	1	0	0	2	4
2. Second distal screw of the plate	4	6	7	8	10	5	6	7	6	5	9	8	10
3. Third distal screw of the plate	10	13	15	18	15	16	14	15	14	13	15	14	13
4. Fourth distal screw of the plate	19	20	21	22	19	20	21	23	20	18	19	18	20

**Table 3:** Distances (mm) between the olecranon fossa and the tips of the four screws at the distal end of the plate in the medial humeral epicondyle

Measurement area	Specimen number/left (L) or right (R) side												
	No. 1 R	No. 2 R	No. 3 L	No. 4 L	No. 5 L	No. 6 R	No. 7 L	No. 8 R	No. 9 L	No. 10 L	No. 11 L	No. 12 R	No. 13 L
1. First distal screw of the plate	6	12	9	2	5	8	5	6	8	10	9	9	0
2. Second distal screw of the plate	1	3	2	1	1	0	0	0	1	2	1	2	3
3. Third distal screw of the plate	0	0	0	0	0	0	1	0	1	0	0	0	7
4. Fourth distal screw of the plate	9	6	5	2	5	1	9	8	8	7	6	5	10

### Clinical case series study

The study was reviewed and approved by the institutional ethics committee, and informed consent was obtained from all patients. Twelve patients with humeral shaft fractures treated with anteromedial MIPPO from 2017 to 2020 were selected. The mean patient age was 53.67 ± 16.60 years (range 26–82 years). There were 6 males and 6 females, 9 cases on the left and 3 cases on the right. All patients were treated with minimally invasive anteromedial LCP fixation. The inclusion criteria were as follows: (1) diagnosis of unilateral closed humeral shaft fracture by imaging examination; (2) no neurovascular injury; and (3) patient consent to undergo surgery. The exclusion criteria were as follows: (1) pathological fracture; (2) associated nerve injury; (3) open fracture; (4) history of mental illness or cognitive impairment; or (5) severe systemic disease resulting in an inability to tolerate surgery.

### Surgical technique

Anesthesia was established by supraclavicular nerve block. The patient was placed in the supine position with 90° arm-to-shoulder extension and forearm supination. The proximal and distal incisions were made as described above, and the plate was inserted distally. By palpation, the spaces between the proximal end of the proximal biceps and the pectoralis major were determined.

**Fig. 8:**
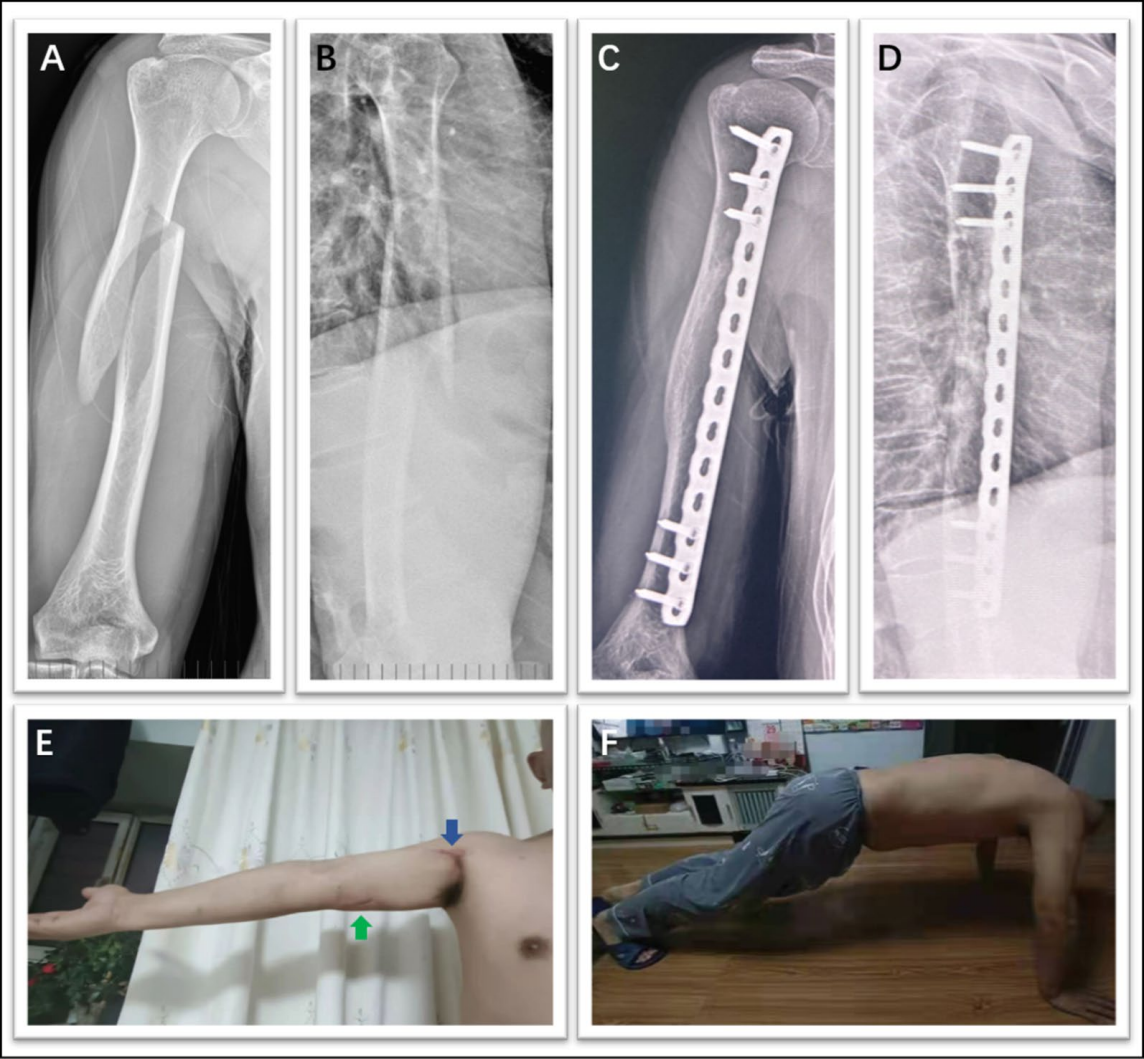
This figure shows a typical case. The patient fell while walking down stairs and sustained a middle fracture of the right humeral shaft. **A**–**B** Preoperative X-rays. **C**–**D** X-rays taken 3 months after surgery, with full recovery of function. **E** Proximal (*blue arrow*) and distal (*green arrow*) incisions. The incision is on the medial side, so the distal scar from the surgical incision is more hidden. **F** Three months after surgery, with full recovery of function

**Fig. 9:**
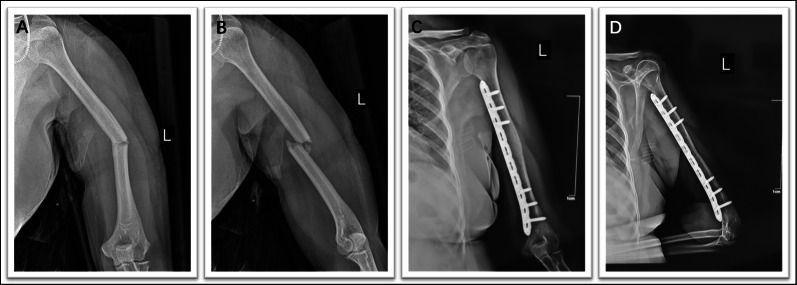
A typical case. The patient fell while walking down the stairs and sustained a middle fracture of the humerus (left side). **A–B** Preoperative X-rays. **C–D** X-rays taken 4 months after surgery

When the arm is extended 90°, the pectoralis major is parallel to the humeral axis. After the skin is cut, the pectoralis major pulls laterally, the long head of the biceps brachii pulls medially, and there is enough space under the long head of the biceps brachii to place the LCP (Fig. 7). Typical cases are shown in Figs. 8 and 9.

### Postoperative management

After surgery, the forearm was suspended for 2 weeks, and the shoulder and elbow joints were moved passively. After 2 weeks, the shoulder and elbow joints were gradually allowed to become active. Strength exercises were performed after X-ray examination showed a bone bridge at the fracture site. None of the patients included in this study developed an iatrogenic nerve injury. X-ray examination was performed within 3 days after the operation, and an outpatient review was performed every 6 months after the operation. The Disability of Arm, Shoulder and Hand (DASH) score was used to evaluate postoperative recovery.

In accordance with the wishes of 1 patient, the internal fixation was purely an elective removed without complications 24 months after surgery. We entered along the original incision and pulled the steel plate from the distal incision. The removal process was smooth and did not cause neurovascular damage; additionally, there were no tissue adhesions that were difficult to remove.

### Clinical observation indexes

The intraoperative blood loss (ml), operation time (minutes), fracture healing time (months), follow-up time (months), and DASH score were evaluated (Table 4).

**Table 4:** Statistical details for the 12 patients

Patient	Age (years)	Gender	Side	AO/OTA type	Plate type	Blood loss (ml)	Intraoperative time (minutes)	Follow-up (months)	Fracture healing time (months)	6 months DASH score	12 months DASH score
1	64	M	L	A1.2	LCP	45	115	16	4	13	5
2	47	F	L	A1.2	LCP	40	97	17	5	12.5	6.6
3	39	M	L	A3.2	LCP	35	93	30	4.5	2.5	1.7
4	52	M	L	A1.2	LCP	40	90	34	3.5	6	2.5
5	46	M	L	A3.2	LCP	35	90	18	3	2.3	1.6
6	65	M	L	A2.2	LCP	30	104	15	4	4	1.6
7	64	F	L	A2.2	LCP	56	96	16	3	12.5	2.5
8	75	F	R	B1.2	LCP	60	93	18	5	6	4.5
9	26	F	R	B1.3	LCP	66	102	16	3.5	2.5	3
10	36	F	L	B1.3	LCP	75	110	17	3.5	6	2.5
11	82	M	L	A3.2	LCP	65	94	16	5	20	9
12	48	F	R	B2.2	LCP	60	85	12	3	5	2.2

*LCP* locking compression plate

### Statistical analysis

IBM SPSS statistics (version 25.0) was used for statistical analysis. Data are presented as the average ± standard deviation (SD).

## Results

### Anatomical analysis

**Fig. 10:**
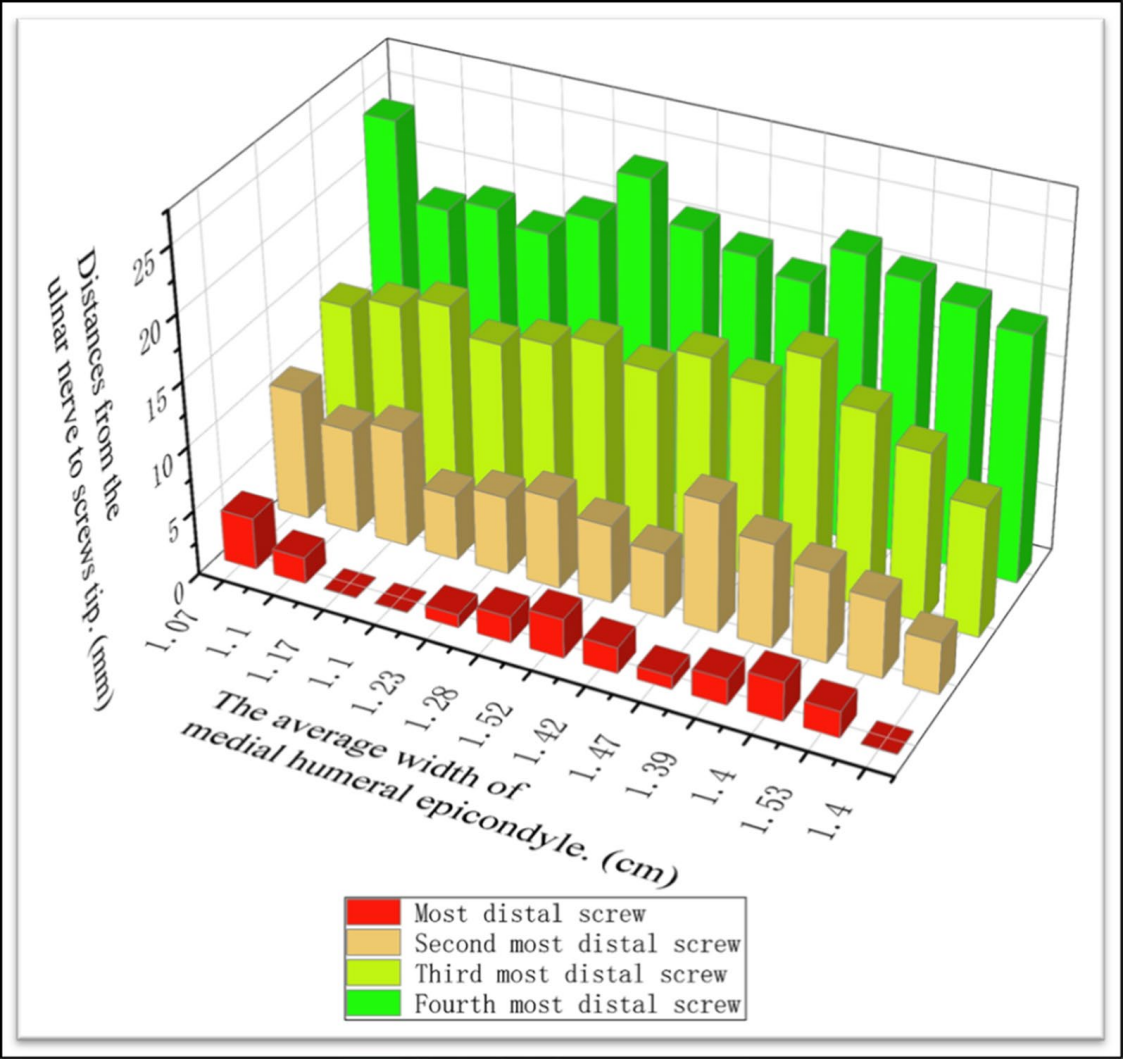
This figure shows the risk of ulnar nerve injury from distal screws. The ulnar nerve could be at risk from the most distal screw

**Fig. 11:**
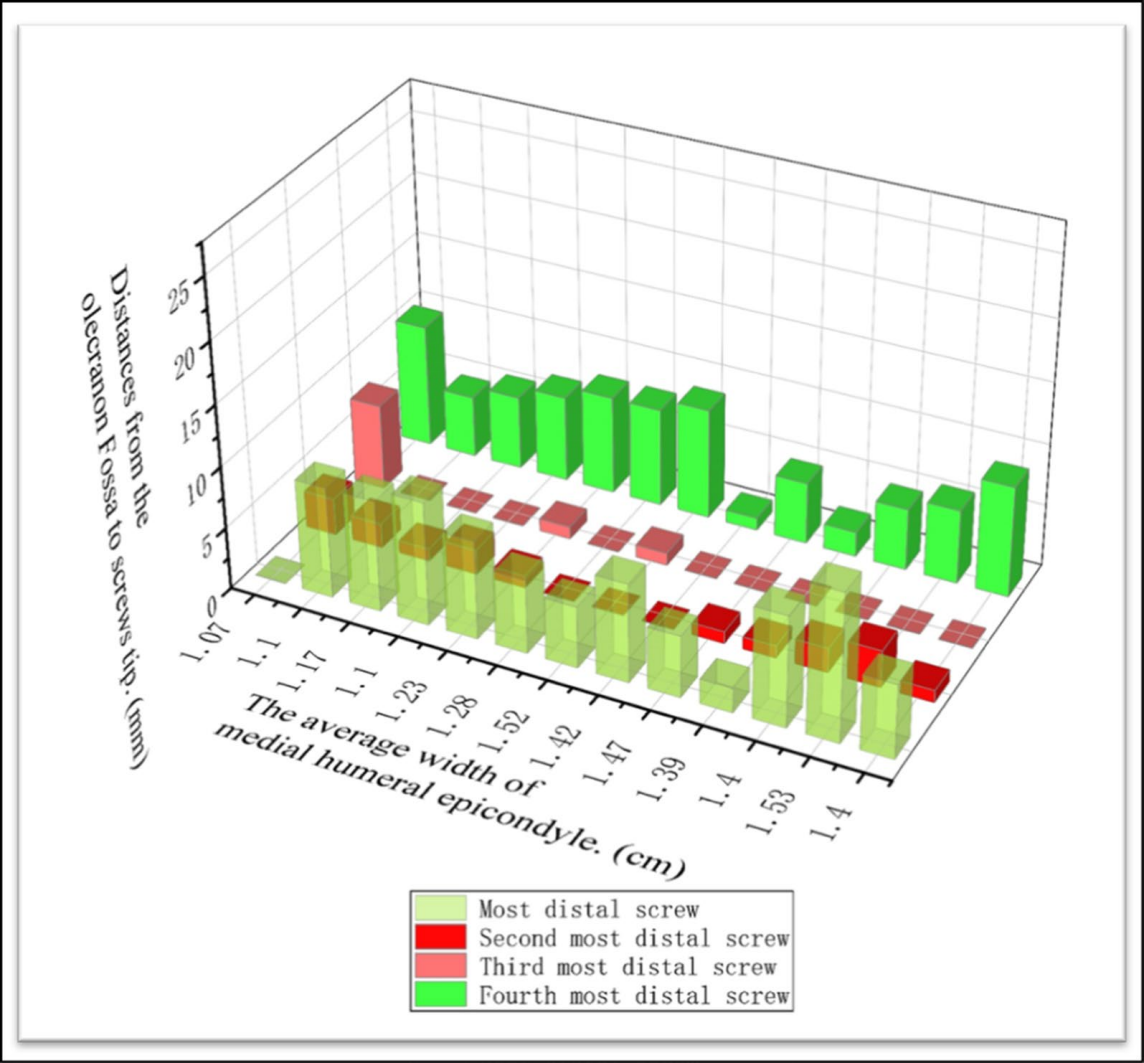
This figure shows the risk of olecranon fossa and articular surface injury from distal screws. The olecranon fossa and articular surface of the olecranon could be at risk from the second and the third distal screws

The average length of the humerus was 29.22 ± 1.62 cm (95% CI 28.24–30.2 cm). The safe area for proximal fixation was 8.88 ± 0.96 cm (95% CI 8.30–9.47 cm) from the anterior medial base of the humeral head to the distal end, and three to four screws could be inserted. The mean distance from the medial condyle to the coronal fossa of the humerus was 1.31 ± 0.18 cm (95% CI 1.21–1.41 cm). The distance from the distal bicortical screw tip of the medial condyle to the ulnar nerve was 2.96 ± 1.62 cm (95% CI 2.79–3.14 cm), and the distance from the medial base of the humeral head to the intersection of the median nerve and the proximal end of the inferior plate was 6.28 ± 0.39 cm (95% CI 6.05–6.52 cm). When the mid-humeral fractures were fixed with an LCP, at least three locking screws could be placed at the distal end, and at least three screws could be inserted into the medial condyle; to avoid injury to the ulnar nerve, the insertion of three screws into the distal cortex is recommended (Table 5 and Figs. 10 and 11).

**Table 5:** Analysis of measurement variability

Measurement area	Range of data	Mean ± SD	95% CI
1. The distance from the medial condyle to the base of the coronal fossa (cm)	1.07–1.53	1.31 ± 0.175	1.21–1.41
2. The vertical distance from the vertex of the medial epicondyle to the median nerve (cm)	2.30–3.40	2.96 ± 1.62	2.79–3.14
3. The length of the humerus (cm)	27.0–32.5	29.22 ± 1.62	28.24–30.2
4. The distance from the medial epicondyle to the lateral epicondyle of the humerus (cm)	5.2–6.78	6.15 ± 0.56	5.81–6.48
5. The distance from the vertex of the epicondyle to the intersection of the median nerve and the distal end of the underlying steel plate (cm)	5.70–6.90	6.28 ± 0.39	6.05–6.52
6. The distance from the medial base of the humeral head to the intersection of the median nerve and the proximal end of the underlying steel plate (cm)	7.00–10.60	8.88 ± 0.96	8.30–9.47
7. Located on the medial epicondyle, the distances between the tips of the bicortical screws and the ulnar nerve, which is crossed by the distal end of the plate with four screws (mm)			
(1). First distal screw of the plate	0.00–4.00	1.70 ± 1.25	0.94–2.45
(2). Second distal screw of the plate	4.00–10.00	7.00 ± 1.91	5.84–8.16
(3). Third distal screw of the plate	10.00–18.00	14.23 ± 1.88	13.10–15.37
(4). Fourth distal screw of the plate	18.00–23.00	20.00 ± 1.47	19.11–20.89
8. Distances between the olecranon fossa and the tips of the four screws at the distal end of the plate in the medial humeral epicondyle (mm)			
(1). First distal screw of the plate	0.00–12.00	6.85 ± 3.31	4.84–8.84
(2). Second distal screw of the plate	0.00–3.00	1.31 ± 1.03	0.68–1.93
(3). Third distal screw of the plate	0.00–7.00	0.69 ± 1.93	−0.47–1.85
(4). Fourth distal screw of the plate	1.00–10.00	6.23 ± 2.68	4.61–7.85

SD *standard deviation*, *95% CI* 95% confidence interval

### Clinical case study

The intraoperative blood loss was 50.58 ± 14.81 ml (95% CI 41.17–59.99 ml), the operation time was 97.42 ± 8.79 min (95% CI 91.84–103.00 min), and the follow-up time was 18.75 ± 6.44 months (95% CI 14.66–22.84 months). The fracture healing time was 3.92 ± 0.79 months (95% CI 3.41–4.42 months), and healing was achieved in all cases. The DASH score was 3.56 ± 2.31 (95% CI 2.09–5.02) at the 1-year postoperative follow-up, with no cases of infection or nosocomial nerve damage. All patients demonstrated fully recovered elbow function at the last follow-up (Table 6).

**Table 6:** Patient descriptive characteristics

Parameter	Range	Mean ± SD	95% CI
1. Age (years)	26.00–82.00	53.67 ± 16.60	43.12–64.21
2. Blood loss (ml)	30.00–75.00	50.58 ± 14.81	41.17–59.99
3. Intraoperative time (min)	85.00–115.00	97.42 ± 8.79	91.84–103.00
4. Fracture healing time (months)	3.00–5.00	3.92 ± 0.79	3.41–4.42
5. 6 months DASH score	2.00–20.00	7.69 ± 5.55	4.17–11.22
6. 12 months DASH score	2.00–9.00	3.56 ± 2.31	2.09–5.02

SD *standard deviation*, *95% CI* 95% confidence interval

## Discussion

The minimally invasive anteromedial approach is a safe and effective technique for the treatment of extraarticular fractures of the middle or distal third of the humerus. There were no complications related to neurovascular injury in the group of patients treated in this study.

Iatrogenic radial nerve injury has been a major complication of anterolateral, lateral, and posterior MIPPO in previous studies. MIPPO has been used to treat middle and distal third humeral fractures of the humerus, leading to a relatively high incidence of postoperative radial nerve palsy of 5.4% [3]. These surgical approaches require exposure of the radial nerve during surgery, which is inconvenient for the surgeon and increases the possibility of iatrogenic radial nerve injury.

Because of the unique anatomy of the distal humerus, for the anterior approach to fractures of the distal third of the humerus, the fracture line needs to be at least 6 cm above the coronal fossa to stabilize the distal bone mass, and the plate is placed anterior to the humerus and close to the coronal fossa to affect the movement of the elbow joint [4]. MIPPO via an anterior approach to the humerus requires the splitting of the brachialis muscle, which may lead to iatrogenic injury to the radial nerve or MCN, resulting in motor weakness [5, 6]. A posterior approach may be selected due to the fracture geometry; this approach also requires the identification and protection of the radial nerves and increases both the difficulty of the surgical procedure and the risk of iatrogenic nerve injury [7, 8].

Some scholars think that fixation at the proximal end of the plate may interfere with the strength of the long head of the biceps tendon and affect the sliding of the biceps tendon [9]. In our cadaveric study, the plate was placed medial to the long head of the biceps brachii, but in clinical practice, the tendon of the long head of the biceps brachii was pulled to the medial side, and the LCP was placed under the long head tendon of the biceps brachii. The positional relationship between the long head of the biceps brachii (LHB) and the LCP differs between cadavers and clinical operations. In a previous anatomical study, due to cadaveric reasons, the LHB was dehydrated and adhered to the surface of the humerus so that the LCP could not be placed under the LHB. In the clinical operation, there was enough space under the LHB to place the LCP. In some patients, the position of the proximal humerus was narrow, so the LCP was placed under the LHB to move the shoulder joint during the operation, and the LCP had no effect on the LHB. The longest follow-up after operation was 3 years, and the shoulder joint activity of the patient was not affected. A recent cadaveric study described an anteromedial MIPPO approach that requires an incision in the pronator teres muscle and the insertion of a steel plate. According to our experience, the fixation device can pull the pronator teres medially during placement on the internal epicondyle of the humerus, and the upper medial condyle of the humerus needs to be completely exposed without cutting the pronator round muscle [10]. Intraoperative ulnar nerve injury is also a concern. In the specimens, the distal bicortical screw tip was very close to the ulnar nerve. To avoid injury to the ulnar nerve and entry of the screw tip into the olecranon fossa, single-layer cortical locking screw fixation is recommended for screws in the medial epicondylar region of the humerus.

The medial approach, as an option for humeral shaft fractures, was first proposed by Judet et al. However, due to the complex anatomical structure of the medial upper arm, this approach is not suitable for open reduction and internal fixation [11, 12]. There have also been a few studies concerning open reduction and internal fixation of the medial humerus [13–15]. These studies have shown that the anteromedial approach is a feasible surgical approach for the treatment of humeral fractures. Moreover, the anteromedial surface of the humerus is flat, and it is not necessary to prebend the steel plate when treating fractures of the middle of the humerus. Our study shows that anteromedial MIPPO can be performed through a soft tissue tunnel under the brachialis muscle without exposing the neurovascular structures of the inner upper arm. This surgical approach carries less risk of iatrogenic radial nerve injury and reduces the risk of muscle weakness caused by anterior MIPPO while affecting the movement of the elbow joint less [16]. Additionally, since the incision is on the medial side, the scar from the surgical incision is more hidden.

The minimally invasive anteromedial approach for the treatment of fractures of the middle and distal thirds of the humerus has some limitations. This clinical therapeutic study included a small number of cases, and this MIPPO technique is not suitable for proximal humeral fractures; there is not enough space proximal for screw fixation, resulting in a smaller amount of humerus available for purchase than in the lateral or anterior-lateral MIPPO approach, which allows the entire humerus/head to be available for purchase.

## Conclusion

The anteromedial MIPPO approach was performed as described, and no iatrogenic neurovascular injury occurred. In the presentation of a novel technique, the results of even a rather small case series might be relevant. This approach can be used as another option for the treatment of extraarticular fractures of the middle and distal thirds of the humerus.

## Data Availability

Not applicable.
